# Introductory Lectures in the Medical Department of Pennsylvania College

**Published:** 1846-03

**Authors:** 


*Introductory Lectures in the Medical Department of Pennsylvania College.*

We have before us the introductories, (in this institution,) to their several courses, of WM. B. GRANT, M. D., Professor of Anatomy, JOHN WITTBANK, M. D., Professor of Midwifery, WM. DARRACH, M. D., Professor of Theory and Practice of Medicine, WASHINGTON L. ATLEE, M. D., Professor of Chemistry; and also the introductory of Professor BEDFORD, of the New York University.

Each of these gentlemen set forth the peculiar claims and relative importance of their respective departments; and each very properly impresses the student with the dignity of his profession, and the necessity of close study and high moral action to preserve this dignity, as well as elevate themselves in their profession.

